# Reporting gaps in immunization costing studies: Recommendations for improving the practice

**DOI:** 10.1016/j.jvacx.2020.100069

**Published:** 2020-07-18

**Authors:** Kelsey Vaughan, Annette Ozaltin, Flavia Moi, Ulla Kou Griffiths, Michaela Mallow, Logan Brenzel

**Affiliations:** a ThinkWell, Washington, DC, USA; b UNICEF, New York, NY, USA; c Bill & Melinda Gates Foundation, Seattle, WA, USA

**Keywords:** Costs, Costing, Methodology, Reporting, Vaccines, Immunization, Immunization economics

## Abstract

High-quality evidence on the cost of delivering vaccines is essential for policymakers, planners, and donors to ensure sufficient, equitable, predictable, and sustainable financing. However, poor practices and reporting oversights in both the published and grey literature limit the understanding and usability of cost data. This paper describes quality assessment results and quantifies problems with immunization costing study reporting practices found in 68 articles and reports included in an immunization delivery unit cost repository focused on low- and middle-income countries and launched in 2018, the Immunization Delivery Cost Catalogue (IDCC). We recommend a standard of practice for writing up an immunization costing study, in the form of an easy to follow checklist, to increase the quality of reporting and the comparability of results. Reporting that adheres to this checklist will improve the comprehension and interpretability of evidence, increasing the likelihood that costing studies are understood and can be used for resource mobilization and allocation, planning and budgeting, and policy decisions.

## Highlights

• Poor practices and reporting oversights limit the understanding and use of immunization cost data.
• Our review identified reporting problems on the vaccines costed, types of costs, and data analysis.
• Our checklist offers a standard of practice for reporting on immunization costing studies.
• Reporting that adheres to this checklist will increase the interpretability and use of evidence.

## 1 Introduction

A number of global trends signal the need for higher quality, more easily accessible evidence about the costs of delivering immunization services, also referred to as immunization ‘delivery costs’ or ‘operational costs’ [1]. Although global immunization coverage remains high, there are still nearly 20 million children under the age of one who do not receive basic vaccines [2]. Most of these 20 million unreached children are in low- and middle-income countries (LMICs) that receive some type of donor support for immunization and health services. As countries transition away from donor support, increased domestic financing for vaccine delivery will be key to achieving and maintaining high coverage rates needed to ensure the population-level benefits of immunization. This is particularly relevant for the 73 countries receiving support from Gavi, the Vaccine Alliance, half of whom have transitioned, are currently transitioning, or are close to entering the final, accelerated phase of transition [3]. Immunization programs are already chronically underfunded [4], [5], and the Learning Network for Countries in Transition (LNCT) predicts that vaccine costs and their associated delivery costs are likely to increase for countries during and after their Gavi transition [6]. At the same time, resource mobilization strategies rooted in updated financial projections and sound fiscal space assessments are required when advocating for funding from the Ministry of Finance and other key decision makers [7].

Until recently, evidence about immunization delivery costs was not easily accessible for use by country immunization managers and policymakers. Cost data were fragmented across organizational websites and peer-reviewed journals with paywalls, or simply not published in the public domain. Released in 2018, the Immunization Delivery Cost Catalogue (IDCC) consolidates the evidence across LMICs on the unit costs of vaccine delivery using a variety of delivery strategies, available at immunizationeconomics.org/ican-idcc.

While cost evidence is now more accessible, the quality of reporting remains an issue. Incomplete reporting is a longstanding shortcoming noted across the field of economic evaluation, and lack of publishing guidance is a recognized problem for global health interventions [8], [9]. Improved reporting has been noted specifically as an area requiring further work for immunization economics [10]. Both methodological differences across studies and inadequate reporting make it hard to know if differences are due to actual variation in costs [11]. From a global perspective, comparing results across costing studies conducted in different countries and contexts is important to identify cost trends and inform high-level policy, such as grant-making from Gavi, as well as to provide a benchmark for policymakers when domestic data is lacking. Standardized reporting (and methods) would help increase the “quality, interpretability and transferability” of cost study findings [12].

Currently, there is no recommended guidance for reporting on immunization costing results to improve the heterogeneity of the quality of articles and reports. Existing immunization-specific methods guidance—either standalone or linked to a costing tool—do not go as far as providing detailed reporting specifications. While extensive guidance exists on reporting on economic evaluations, the costing aspects of this guidance are not sufficiently detailed and often refer to secondary cost data as opposed to primary data which are frequently used in immunization and other health services costing studies in LMICs and carry unique reporting requirements [13], [14]. The Global Health Cost Consortium’s (GHCC) Reference Case, released in 2018, provides guidance for reporting costing study findings related to HIV and TB programs [8]. The 17-point principles and methods checklist is organized around study design and scope, service and resource use measurement, valuation and pricing, and analyzing and presenting results. While a useful tool with many relevant items for costing studies which fall outside the domain of HIV and TB programs, the reference case does not cover all relevant reporting needs for immunization program cost results. Notably, specifics about the vaccine(s) costed and guidance on common terminology used in the immunization costing community are understandably missing from the GHCC Reference Case.

This paper outlines the methods for assessing the quality of articles and grey literature included in the IDCC, categorizes and quantifies the main reporting deficiencies found, and provides a reporting checklist specific to immunization costing for researchers and practitioners who are writing up primary costing study findings from LMICs, whether it be for published or grey literature.

## 2 Methods

We conducted a systematic review on immunization costs with a search that included 17,439 peer-reviewed articles and grey literature reports from January 2005 to March 2019, from which we ultimately extracted data from 68 articles and reports (the sample). Systematic review methods, summary findings, and the IDCC repository are discussed elsewhere [15], [1].

During the review process, we conducted a quality assessment of the included articles and reports, which identified gaps and poor practices in reporting according to reporting and scope of the evidence. For reporting, we built on existing quality assessment systems and checklists to evaluate each article or report in three areas: methodological rigor and reporting standards (10 items), possible uncertainty of results (3 items), and risk of bias and limitations (2 items) [16], [17], [18], [19], [13], [20], [8]. This review on reporting included 64 of the 68 articles and reports in the sample; four did not undergo quality assessment: one was a dataset and personal communication with the EPIC Immunization Costing team, while for three others the review team participated in the writing of the grey literature reports, introducing a conflict of interest. Each of the 15 quality assessment items was given an individual score of 1 (lowest), 2, or 3 (highest); for some items there was also a “not applicable” option. Scores for all items were summed and averaged, excluding any ‘‘not applicable” answers, to produce a final score for each resource on the 1–3 scale.

For scope, we counted the unit cost dataset (IDCC, n = 68) to identify the number of articles and reports meeting different reporting criteria (e.g., number including paid human resources as a cost category, number identifying the types of costs included in each unit cost estimate, etc.). Based on these counts, we described the evidence and identified gaps.

We also attempted to identify commonalities between, for example, study publication date or study country, and reporting problems. We did not observe any such clustering of reporting problems so we have excluded this from our results.

We describe the results of both reviews by categorizing and quantifying the reporting problems in four areas: (1) Vaccines costed and their delivery; (2) Types of costs included; (3) Data analysis; and (4) Other study design and methodology issues.

## 3 Results

### 3.1 Quality scoring

The quality review of the 64 articles and reports in the sample gave an overall mean score of 2.3/3.0 (Table 1). There was little difference in mean score between the three quality assessment areas, with each area scoring an average of 2.3/3.0. An overwhelming majority of the sample did not report sufficient methodological detail on the data analysis strategy, including use of statistical methods and confidence intervals (1.2/3.0). Sensitivity testing also scored poorly (1.3/3.0), despite sensitivity analysis being a widely recommended methodological best practice for economic evaluations, where costing is one component [18]. Contextual factors and the purpose of the study were most consistently reported (3.0/3.0).

**Table 1:** Immunization Costing Action Network (ICAN) Quality Assessment Results (n = 64 articles and reports).

Quality Attribute		Mean Score (low to high scale from 1 to 3)
**Methodology and reporting**		**2.3**
	Quality of input data/data sources	2.5
	Sample strategy in relation to conclusion and generalizability	2.7
	Data analysis strategy	1.2
	Allocation of shared costs	1.8
	Annualization of capital items	1.9
	Replicability: methods	2.2
	Replicability: study purpose	3.0
	Reporting of results	2.4
	Accuracy of reported findings 1:Does sum of capital and recurrent items match total?	2.8
	Accuracy of reported findings 2: Does sum of cost categories match total?	2.6
**Uncertainty of results**		**2.3**
	Sensitivity analysis	1.3
	Missing cost categories	2.6
	Contextual factors	3.0
**Risk of bias/limitations**		**2.3**
	Author-stated limitations	2.2
	Extractor-perceived limitations	2.4
**Overall total**		**2.3**

### 3.2 Reporting gap: Vaccines costed and their delivery

We identified a number of issues related to reporting on the vaccines costed. In some cases, authors noted they costed the *full schedule* of vaccines, but failed to note which vaccines were included in the schedule. In other cases, they identified the vaccine simply as pneumococcal vaccine (PCV) or human papillomavirus vaccine (HPV), without providing any information on the type (e.g., PCV10, bivalent HPV), manufacturer, presentation (i.e., number of doses per vial versus pre-filled syringe) and required storage space and packaging of the vaccine. These details are important because vaccines represent the largest share (approximately 50%) of total immunization program cost, but the cost can vary substantially depending on these vaccine characteristics [15], [21].

Vaccine delivery details, target population, and other related contextual information are also important, but frequently not reported. For example, 43% of the sample omitted information on the number of doses delivered within the period of study, information which gives you a sense of the cost-volume relationship and is needed for calculating some unit costs. Thirty percent of the sample did not state the types of facilities where immunization was provided and analyzed for the costing study (e.g., public facilities, private, NGO). Finally, 25% of the articles and reports excluded information on the target population. For some vaccines, such as Bacille Calmette-Guerin (BCG) vaccine given to newborns, one can deduce the target population, but for others it is not as clear (e.g., oral cholera vaccine (OCV) delivered through a campaign may target children and adults of different ages).

### 3.3 Reporting gap: Types of costs included

We found inconsistent practices in reporting on types of immunization costs. For 12% of the sample, it was not stated whether costs were valued economically, financially or fiscally. We also found variations in the types of immunization program cost categories used and their naming. For example, the terms “administration”, “program management”, “program administration”, “overhead” and “planning” were seemingly used interchangeably by different authors to describe costs related to managing the Expanded Programme on Immunization (EPI), though the individual cost items included in these categories were not specified and may have differed. Several cost categories were frequently not reported, but were not explicitly noted as being excluded either (i.e., per diem and travel allowances; program management; disease surveillance and activities related to adverse events following immunization; buildings, utilities, other overheads and/or shared costs; and waste management). Some articles and reports stated costs by activity, such as outreach service delivery or surveillance, while others reported line items such as paid human resources and transport. Still others combined activities and line items: reporting on vaccine transport (line item), staff (line item), training (activity), and implementation (activity).

### 3.4 Reporting gap: Data analysis methods

Reporting on data analysis methods was found to be a major gap. Important analytic techniques, such as how data were aggregated across facilities or extrapolated beyond the sample to arrive at a national cost estimate, were not reported in 19% of the sample. Recent research has shown wide variation in the results according to methods used in multi-site healthcare costing studies in LMICs [22], making this a critical item for analysis and reporting.

Eighteen percent of articles/reports in the sample did not report the currency year, and 47% did not report methods for converting the original currency to the reported currency (e.g., converting from 2016 Ugandan shillings to 2019 U.S. dollars). Reporting on methods for allocating shared costs and annualizing capital items was also frequently missing (in 53% and 35% of the sample, respectively). Other authors did not report the total cost (32% of the sample), number of doses delivered and/or children immunized (44%), making it difficult to understand how unit costs were estimated. Finally, although a widely accepted standard best practice [18], 65% of the sample did not report any sensitivity analysis on delivery cost variables for which there is uncertainty.

### 3.5 Reporting gap: Other study design and methodology issues

The methods descriptions in 66% of the sample were not sufficient to allow another researcher to replicate the study. Fifteen percent of sampled articles and reports did not name or describe their data sources.

The sampling frame and sample size are essential reporting items, as size of the sample and how it was drawn help determine the representativeness and generalizability of the study. However, 18% of the sample did not state the number of sampled facilities, and 28% either did not describe the sampling strategy or described it in an insufficient manner.

## 4 Immunization costing study reporting: A checklist of key items to include

The identified reporting deficiencies make it difficult for the reader to assess the quality of the findings and therefore limit their use. Additionally, these omissions and reporting differences may or may not drive differences in results. Specific and structured guidance on how to write up a costing study is a critical need in the field of immunization economics to facilitate more comprehensive reporting on methods by authors. This will allow readers to evaluate studies on their robustness and scientific rigor, and better compare results of different studies.

We utilized the GHCC Reference Case checklist (summarized in Table 2) as a starting point for the development of an immunization costing reporting checklist. We deleted items which were irrelevant (e.g. specification of longitudinal versus cross-sectional data and patient sampling) and incorporated additional items (e.g. information about the vaccine(s) costed and their delivery), and also provided specific text recommendations for responses relevant for immunization costing in the “Reporting Options” column (Table 3).

**Table 2:** Global Health Cost Consortium (GHCC) Reporting Checklist Items.

Checklist Items	Topics
Study design and scope	Study purpose, population, intervention/service/output, perspective, type of cost, units, time horizon
Service use and measurement	Definition of cost categories included, inputs and their quantities, data sources, sampling strategy, data collection timing
Valuation and pricing	Data sources, annualization/depreciation of capital items, discount rate, inflation, exchange rate, opportunity cost
Analyzing and presenting results	Variations by subgroups, uncertainty, bias, limitations, conflicts of interest

Source: adapted from [8].

**Table 3:** Immunization Costing Reporting Checklist.

Checklist Item	Reporting Options*
***Study design and scope***	
Study type	Example responses: Costing study, economic evaluation, financial planning, budget impact analysis, efficiency analysis, other

Aim of the cost analysis	Free text; may be useful to include a description of the process undergone to arrive at the aim, including stakeholders (target audience) and how they were engaged

Relevance for health practice and/or policy decisions	Free text; address any relevant parameters of practice and/or policy that informed the conceptualization and design of the study

Vaccines costed	Recommended sub-items: Manufacturer, trade name, presentation (doses/vial or pre-filled syringe), packaging and packed volume (cm3/dose) Schedule of required doses and timing Unit price per dose Total doses delivered at study sites (or other totals used for extrapolation beyond the sample)

Target population	Recommended sub-items: Age, sex, geographical location, etc. of those targeted for vaccination

Coverage level	Recommended sub-items: Coverage of costed vaccine as percentage of target population at sites For costing of multiple vaccines, overall program coverage
Delivery strategy and sector	Recommended sub-items: Health facility, outreach/mobile, school, campaign, national immunization days/weeks or child health days/weeks, along with description of the strategies Routine or supplemental immunization activity (SIA) Public, private or NGO

Study perspective	Example responses: Government, provider, health system, societal, other (may include military, schools, etc.). Free text to define the perspective with clarification on disbursing agents or the ultimate sources of funds

Retrospective costing vs. cost projection	Example responses: Retrospective costing of an existing immunization intervention vs. cost projection for introduction of a new vaccine

Economic vs. financial vs. fiscal cost	Example responses: Economic vs. financial vs. fiscal cost (see Box 1)

Incremental cost?	Yes/no

Period type (start-up vs implementation)	Example responses: Introduction/start-up, recurring/ongoing, or both periods included in the costing If reporting introduction/start-up costs, clearly define the start and end points (with dates) for the start-up/introduction period, the included cost categories for each period, and lifetimes and discount rate for annualizing introduction costs

Costs included from which level(s) of the health system (i.e. where costs were incurred, though funding may come from different/higher levels)	Example responses: Facility, district, province/region, national, outside the country setting (e.g. international, externally-funded technical assistance). Note if any levels of the health system were explicitly excluded

***Service use and measurement***	
Cost categories included	Recommended sub-items: Cost categories included (specify line items separate from activities) Cost category definitions Justify any cost category exclusions

Sampling frame, methods and size	Recommended sub-items: Sampling frame Sampling methods Sample size (communities if applicable, facilities, districts, provinces/regions)

Timing of data collection	Dates of data collection

Measurement approach for each input	Example responses: Top down/budget or expenditure report extraction, bottom-up/ingredients-approach, or free text

Data source used to measure the units (source, method and allocation/tracing factors to allocate shared resources, such as building space, equipment, human resources, vehicles)	Example responses: Data sources: Desk/record review (immunization records, expenditure reports, etc.), interviews, observation, post-introduction survey, other Method: structured questionnaires, topic guides, etc. Note how tools were designed, tailored and tested Allocation/tracing factors: Interview, observation, time sheets, floor space, vehicle use logs, other

***Valuation and pricing***	
Data sources used for unit prices	Example responses: Desk/record review (budgets, expenditure reports, procurement invoices, etc.), interviews, other For costs incurred outside country setting: note if costs were valued using international or domestic prices

Methods for valuing volunteer time	Free text

Methods for valuing donated or subsidized goods	Free text

Depreciation approach, useful lifetimes and discount rates for capital items	Example responses: Approach: straight line depreciation, amortization Lifetimes: 10 years, 15 years, etc. and source Discount rates: 3%, 5%, etc. and source

Currency	Recommended sub-items: Currency and year

Any currency conversions made	Recommended sub-items: Exchange rate, source and year

Inflation type and rate used	Example responses: Percentage, GDP deflator/CPI, source

***Analyzing and presenting results***	
Definitions of unit costs used and how they were calculated (numerator and denominator)	Recommended sub-items: Cost per: dose, capita, person in the target population, fully immunized child, full immunization of a vaccine, other unit costs (such as cost per cubic meter for supply-chain related studies)

Analytical techniques used	Recommended sub-items, if applicable, with free text response: Aggregation Generalizing costs beyond the data collected either in terms of time or geography (specify time periods used for generalizing costs beyond the time period collected) Confidence intervals Multivariate statistical methods used to analyze cost functions Statistical methods used to establish differences in unit costs by sub-group Methods for imputing missing data and extent of the problem

Any scenarios or assumptions used for cost projections [if applicable]	Free text

Relevant total and unit costs	Recommended sub-items: Report total costs with time period noted (e.g. one year) Report unit costs with and without the vaccine price included and with and without paid human resources Note percentage share of each cost category of the total and unit cost

Any sub-groups or populations analyzed	Free text

Sensitivity analysis findings	Free text

Possible sources of bias, limitations in the design, analysis, results and conclusion	Free text; include data challenges and analytical techniques used that may bias results and conclusions as well as aspects of the cost estimates that would limit generalizability of results to other contexts

* Depending on the Checklist Item, the Reporting Options column may include example response options, recommended reporting sub-items or further prompts to guide reporting.

Where possible, all items should be reported in the main body of the report or publication. Where space does not allow, the full checklist can be included in an annex or supplemental file. Key parts of the checklist are described below.

### 4.1 Study design and scope

Study design and scope includes the study purpose, target population, perspective, type of cost, vaccines costed and time horizon. For immunization costing studies, this means clearly reporting the purpose of the study and any context for the study that will improve use and understanding, for instance, whether the costing exercise was done in the context of an economic evaluation, financial planning activity, policy input, or programmatic evaluation. The relevance of the results for health practice and/or policy decisions should be noted, along with any relevant parameters of practice and/or policy that informed the conceptualization and design of the study.

The vaccine(s) costed, their target population(s), coverage level(s), delivery strategy and sector should be discussed. Essential details about the vaccine(s) costed include manufacturer, trade name, presentation, packaging and packed volume, unit price per dose and the total number of doses delivered. An example might be Bivalent Human Papilloma Virus (Types 16 and 18) vaccine – 2 dose vial in cartons of 100 vials, cold chain volume 4.8 cm^3^ per dose. GlaxoSmithKline Biologicals. $4.50/dose [23] for 278,291 total doses delivered. Because not all vaccines require the same number of doses (e.g., Rotavirus 2- and 3-dose vaccines), and schedules vary by country, the number of doses per vaccine and their timing should be clearly specified. When multiple vaccines or a full schedule are costed, all vaccines included in the costing should be noted. The total number of doses delivered at the study sites (or totals used for extrapolation beyond the sample) should also be reported, as well as any coverage levels assumed. Target population should be named where it is not obvious. Authors should note the delivery strategy employed, whether delivery was in the context of routine immunization or a supplemental immunization activity (SIA), such as a campaign, and whether delivery occurred via the public, private and/or NGO sectors.

Authors should state whether a government, health care provider, or societal perspective was taken. This should be determined based on by whom the costs were incurred [24], but because of some confusion around the concept in general, as costs incurred can be interpreted as disbursing agents or the ultimate sources of funds (e.g. funds disbursed by the Ministry of Health but provided by a donor organization) [8], the perspective should also be defined. Reporting should also note whether the costing study was a retrospective costing of a past or existing immunization intervention or program, or an estimate of future program costs related to introduction of a new vaccine or change in delivery strategy.

Box 1 **Definitions of Economic, Financial, and Fiscal Costs.**

**Table:** 

**Economic costs**: Financial outlays plus opportunity costs of health worker time and any donated items such as vaccines. Depreciation of capital items using a discount rate.
**Financial costs**: Financial outlays, usually with straight-line depreciation of capital items.
**Fiscal costs**: Financial outlays, usually without depreciation of capital items.
Source: [1], adapted from [27].

Authors must clearly define whether the costs are economic, financial or fiscal (Box 1). Incremental costs should be clearly identified, and authors should indicate if costs are introduction/start-up costs (generally for a new vaccine introduction), recurrent/ongoing costs (for routine implementation), or a combination of both. If reporting introduction/start-up costs, clearly define the start and end points (with dates) for the start-up/introduction period, the included cost categories for each period, and lifetimes and discount rate for annualizing introduction costs.

The reporting should indicate at which level(s) of the health system costs were incurred (e.g., facility, district, province/region, national, outside the country setting).

### 4.2 Service use and measurement

Authors should report the sampling frame and method, as well as the sample size (facilities, districts, provinces/regions). Number of facilities may be omitted in favor of number of communities or inhabitants, such as for campaigns. Authors should also describe how respondents were selected (i.e., sampled, convenience) for interviews, if this technique was used for data collection. The timing of data collection should also be reported.

### 4.3 Valuation and pricing

This checklist item comprises data sources, annualization/depreciation of capital items, discount rate, inflation rate, exchange rate and opportunity cost. Authors should detail methods so that the study could be replicated by others. This means specifying the measurement approach used for each input (frequently either top-down via budget or expenditure report extraction or bottom-up using the ingredients approach) and the data sources for both quantities and prices.

### 4.4 Analyzing and presenting results

Included in this checklist category are all issues related to analysis and results, including variations by subgroups, uncertainty, bias, limitations and conflicts of interest. In terms of analysis, the use of analytical techniques, such as methods used to fill missing data, aggregate, generalize costs beyond the sample, as well as statistical methods to generate confidence intervals, sub-group analyses and sensitivity analyses (if appropriate given the data and policy question), should be reported. Any data quality issues should be clearly noted, along with the extent of such issues and any analytic methods employed to address them.

In terms of results, specificity is critical in reporting the findings of immunization costing studies, given the range in types of costs (economic/financial/fiscal). It is highly recommended that researchers report some combination of cost per dose, cost per capita, cost per full immunization of a vaccine, cost per fully immunized child (FIC), and cost per person in the target population. Full immunization of a vaccine refers to a completed series (all required doses of a specific vaccine (e.g., two doses of oral cholera vaccine (OCV)), while FIC refers to the provision of required vaccines to a target population by a clear point in time (e.g., infants who received all doses of a vaccine in the schedule before reaching one year of age). The definition of FIC utilized in the analysis should be clearly reported. Authors should make it clear how total or unit costs were calculated; any denominators (e.g., number of doses delivered, FICs, population, and/or persons in the target population) used to estimate unit costs should be noted. State the type of cost, year and currency for all total and unit costs.

Recommended best practice is to report unit costs with and without the vaccine costs included, as the vaccine price may differ by setting or change over time. Additionally, we recommend reporting unit costs with and without paid human resources, as human resources are generally financed from a separate budget than the immunization program. Further, it is best practice to report the percentage share of each cost category of the total and unit costs.

If a sensitivity analysis was done, results should be presented as part of the findings, and if not done, the rationale specified. Finally, any limitations, bias or conflicts of interest should be noted.

## 5 Conclusion

This paper provides much needed immunization-specific guidance for reporting on immunization costing studies, building on the seminal reference case developed for global health. Our recommendations are rooted in the review of 68 articles and reports undertaken as part of the IDCC systematic review, which shed light on common shortcomings in the reporting of immunization costing studies in LMICs. As part of the systematic review, we contacted authors to obtain and clarify missing or unclear key information, but the typical reader should not have to make this effort to understand basic study details.

Our immunization-specific checklist aims to increase the comprehensiveness and consistency in reporting of immunization costing studies, by providing a list of essential items to be included, along with sample text for reporting. Improved reporting may facilitate better understanding and correct use of cost findings for resource mobilization and allocation, planning and budgeting, and policy decisions, though likely other factors also contribute to cost evidence not being used in these processes [25]. Additionally, greater consistency and detail in reporting the methodology of costing studies can facilitate cross-study comparison of findings, offering insights into global trends and potentially providing policymakers with reasonable cost benchmarks when national data are lacking.

Ongoing work led by the World Health Organization aims to identify and agree on gaps in current immunization costing resources (i.e., methodological guidance, tools) and priorities for harmonization of terminology, definitions and methods, which will further equip researchers and practitioners with the guidance they need for excellence in reporting.

## Declaration of Competing Interest

The authors declare that they have no known competing financial interests or personal relationships that could have appeared to influence the work reported in this paper.
